# Low‐Temperature Photocrystallization of Atomic Layer Deposition‐Processed Tin Oxide for Highly Efficient and Flexible Perovskite Solar Cells

**DOI:** 10.1002/smsc.202500196

**Published:** 2025-07-07

**Authors:** Dayeon Ko, Se Hun Joo, Sol Kim, In Soo Kim, Minwoo Park

**Affiliations:** ^1^ Department of Chemical and Biological Engineering Sookmyung Women's University Seoul 04310 Korea; ^2^ Nanophotonics Research Center Korea Institute of Science and Technology (KIST) Seoul 02792 Korea; ^3^ KIST‐SKKU Carbon‐Neutral Research Center Sungkyunkwan University (SKKU) Suwon 16419 Korea

**Keywords:** atomic layer deposition, flexible perovskite solar cells, photocrystallization, tin oxides, ultraviolet light

## Abstract

Atomic layer deposition (ALD) enables an excellent surface coverage and uniformity in the preparation of large‐area metal‐oxide thin films. In particular, ALD‐processed SnO_2_ has demonstrated great potential as an electron transport layer in flexible perovskite solar cells (PSCs) and tandem modules. However, the poor electrical conductivities and surface wettabilities of amorphous SnO_2_ remain critical challenges for commercialization. In this study, a low‐temperature and rapid crystallization process for amorphous SnO_2_ is introduced, based on the use of high‐power ultraviolet (UV) exposure (UV‐SnO_2_) to achieve high‐performance flexible PSCs. The generation of highly dense O_3_/OH radicals under UV exposure effectively ruptures the imperfect and weak bonds in the SnO_2_ matrix, thereby facilitating the formation of nanocrystalline SnO_2_. This transformation enhances the conductivity and shifts the energy levels upward, promoting electron injection and transfer from the perovskite. Rigid and flexible devices exhibit remarkable power conversion efficiencies (PCEs) of 22.86 and 21.49%, respectively. Furthermore, the flexible device demonstrates an excellent mechanical durability and environmental stability, retaining 93.3% of its initial PCE after 1500 bending cycles (*r* = 12 mm) and 87.4% after 1000 h under 1 sun illumination. These results highlight the potential of photocrystallization for advancing flexible PSC technologies.

## Graphical abstract

Photocrystallization of ALD‐processed SnO_2_ thin films is successfully achieved at a low temperature. High‐power UV exposure crystallizes amorphous SnO_2_ matrices within 15 min, enhancing electrical conductivity and band alignment with perovskite. This process enables the fabrication of highly reproducible and flexible perovskite solar cells with an impressive power conversion efficiency of 21.49%.

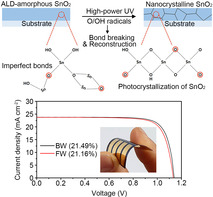

## 1 Introduction

Organic–inorganic halide perovskites are widely used in various semiconductor devices, including solar cells, light‐emitting diodes (LEDs), photodetectors, and transistors.^[^ ^1^, ^2^, ^3^, ^4^ ^]^ In addition to their exceptional optoelectrical properties, the intrinsic flexibility of these materials has garnered considerable attention for the preparation of deformable electronics. Unlike metal oxide perovskites, secondary interactions between tailored organic cations significantly reduce the Young's modulus (5–20 GPa), giving a value comparable to those of organic materials.^[^ ^5^, ^6^, ^7^ ^]^ This unique mechanical property facilitates the fabrication of high‐performance flexible perovskite solar cells (PSCs). In PSCs, the charge transport layer (CTL) plays a crucial role in determining both device performance and mechanical stability. The CTL should have the capability to retain efficient charge transfer under mechanical stress. Vacuum‐processed metal oxide thin films meet these requirements, offering excellent charge transport capabilities and surface coverage without undergoing mechanical failure.^[^ ^8^, ^9^, ^10^ ^]^ In this context, atomic layer deposition (ALD) has emerged as a powerful technique for designing high‐quality flexible CTLs, wherein its ability to prepare pinhole‐free, conductive, and smooth thin films leads to significantly enhanced performances in large‐area devices.^[^ ^11^, ^12^, ^13^ ^]^ Recent PSCs incorporating ALD‐processed SnO_2_ electron transport layers (ETLs) have achieved remarkable power conversion efficiencies (PCEs) exceeding 23%.^[^ ^14^, ^15^, ^16^ ^]^ In addition to single heterojunction cells, the application of ALD has been expanded to the fabrication of perovskite‐based tandem cells. For example, a flexible Cu–In–Ga–Se/perovskite tandem cell demonstrated efficient electron extraction and transfer capabilities through the SnO_2_ ETL on the perovskite layer, achieving an impressive PCE of 23.64%.^[^ ^17^ ^]^ This advancement paves the way for the commercialization of flexible PSCs.

However, critical challenges remain regarding the poor stabilities and electrical properties of ALD‐processed amorphous SnO_2_ in n‐*i*‐p structured devices.^[^ ^18^ ^]^ In general, the preferred precursor is tetrakis(dimethylamino)tin(IV), which leads to the formation of amorphous SnO_2_, as observed in other ALD‐processed metal oxides synthesized using similar precursor chemistry.^[^ ^19^, ^20^, ^21^ ^]^ The resulting amorphous SnO_2_ phase, characterized by randomly coordinated Sn and O atoms, exhibits highly energetic and unstable surfaces that are prone to rapid contamination by atmospheric carbon impurities.^[^ ^22^, ^23^ ^]^ In addition, the reduced surface energy of SnO_2_ can hinder wetting by the perovskite solution, thereby reducing the reproducibility of the device fabrication process.^[^ ^22^, ^23^ ^]^ Furthermore, amorphous SnO_2_ exhibits a poor electrical conductivity, which stems from insufficient oxygen defects (*O*_def_) at low temperatures, thereby limiting the electron transfer efficiency.^[^ ^24^ ^]^ To address these limitations, the additional deposition of SnO_2_ nanocrystals onto ALD‐processed SnO_2_ ETLs has been introduced, demonstrating an enhanced stability and a superior device performance.^[^ ^15^, ^16^ ^]^ However, controlling and modifying the intrinsic properties of the ETLs involved in defect and conductivity management require alternative approaches, such as plasma‐assisted ALD or post‐thermal annealing.^[^ ^25^, ^26^ ^]^ These methods necessitate annealing temperatures of >180 °C, which can damage plastic substrates and pose a significant challenge to the fabrication of flexible devices. The studies into the crystallization of amorphous metal oxides using short‐wavelength light sources have provided protocols for the low‐temperature synthesis of highly efficient ETLs on plastic substrates.^[^ ^27^, ^28^ ^]^

**Figure 1:**
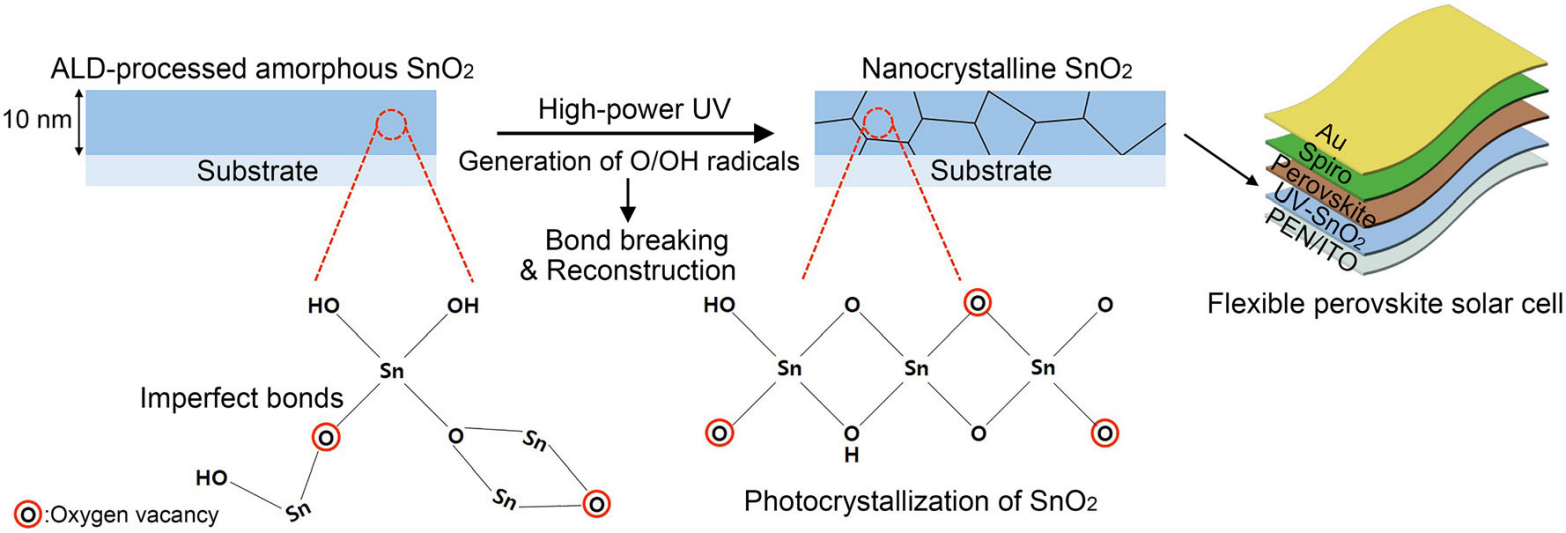
Schematic illustration of the photocrystallization of ALD‐processed amorphous SnO_2_ thin films under high‐power UV exposure. These films serve as the ETLs in both rigid and flexible perovskite solar cells.

With these considerations in mind, this study reports the use of high‐power ultraviolet (UV) light (intensity = 400 mW cm^−2^) to crystallize ALD‐processed SnO_2_ ETLs (UV‐SnO_2_) at low temperatures. **Figure** **1** shows a schematic representation of the radical‐assisted crystallization of 10 nm‐thick SnO_2_ ETLs. Notably, the power of the UV light employed in this system is ≈5–20 times higher than those of commercial UV–ozone generators. Such intense UV exposure is expected to decompose atmospheric oxygen and water molecules into reactive O_3_/OH radicals, assisted by the photocatalytic activity of SnO_2_. Upon exposure, these radicals have the potential to readily break imperfect and weak atomic bonds, which in turn can lead to the formation of new chemical bonds and the creation of O_def_. Reconstruction of the SnO_2_ matrix via such a mechanism is investigated, and its role in promoting the formation of crystalline phases with enhanced stabilities and electrical conductivities is evaluated. Subsequently, the prepared SnO_2_ ETLs are successfully applied to both rigid and flexible substrates, and their corresponding PCEs are measured.

## 2 Results and Discussion

**Figure 2:**
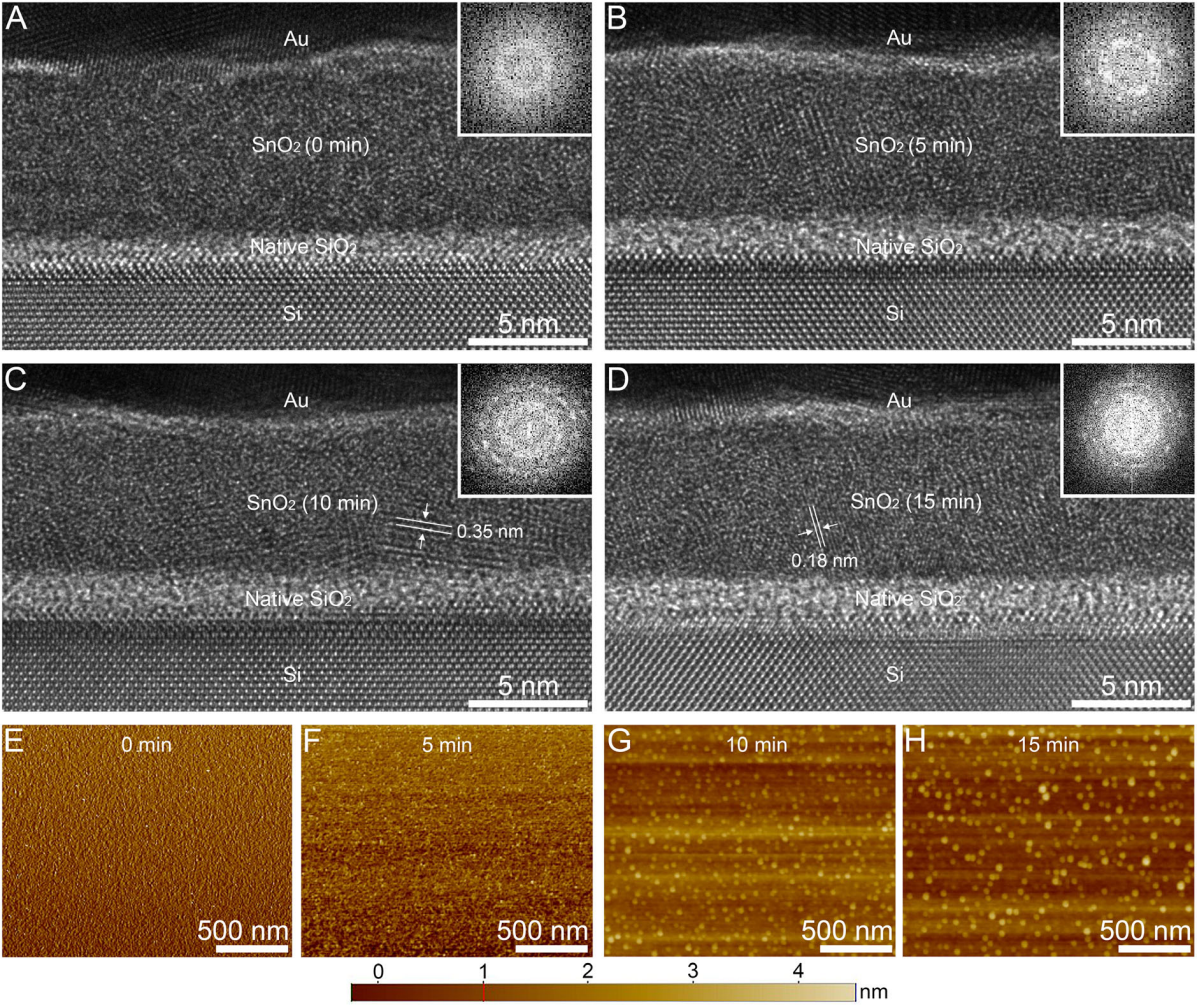
Cross‐sectional TEM images of A) UV0‐SnO_2_, B) UV5‐SnO_2_, C) UV10‐SnO_2_, and D) UV15‐SnO_2_. AFM images of E) UV0‐SnO_2_, F) UV5‐SnO_2_, G) UV10‐SnO_2_, and H) UV15‐SnO_2_. The insets in panels (A–D) show the FFT electron diffractions of SnO_2_.

Based on the secondary photochemical reaction, the photodissociation of oxygen molecules is promoted by the photocatalytic effect of SnO_2_ surfaces under UV exposure.^[^ ^29^, ^30^, ^31^, ^32^ ^]^ The resulting O_3_ molecules subsequently decompose atmospheric water into OH radicals. The theoretically calculated concentrations of OH radicals at relative humidities (RHs) of 30% and 50% are 6.3 × 10^8^ and 9 × 10^8^ molecules cm^−3^, respectively (calculation details in Supporting Information). These highly concentrated free radicals can break metal–oxygen bonds. Previous studies on the photocrystallization of amorphous TiO_2_ and ZnO support the rearrangement of metal–oxygen bonds through bond cleavage under UV exposure.^[^ ^27^, ^28^ ^]^ In the case of SnO_2_, OH radicals possess sufficient enthalpy (1600 kJ mol^−1^) to readily cleave Sn—O bonds (bond strength = 548 kJ mol^−1^), leading to the formation of oxygen vacancies and interstitial oxygen species within the SnO_2_ matrix.^[^ ^33^, ^34^ ^]^ The resulting reconfiguration of dissociated Sn and O sites is accompanied by the crystallization of the amorphous phase. **Figure** **2**A–D presents the cross‐sectional transmission electron microscopy (TEM) images of the SnO_2_ ETLs after UV exposure. In the pristine SnO_2_ ETL (UV0‐SnO_2_), atoms were randomly coordinated without a specific orientation, and no fast Fourier transformed (FFT) electron diffraction was observed (Figure 2A, inset). The central ring indicates the diffused electron scattering by the disordered SnO_2_ matrix.^[^ ^35^ ^]^ After 5 min (UV5‐SnO_2_), randomly oriented crystalline lattices appeared, along with electron diffraction spots (Figure 2B, inset). After 10 min (UV10‐SnO_2_), most regions comprised individual SnO_2_ grains, and electron diffraction rings were distinctly visible (Figure 2C, inset). The indicated *d*‐spacing of 0.35 nm corresponds to the (110) plane of the rutile tetragonal structure. Notably, further UV exposure did not cause any significant changes in crystallinity. After 15 min of exposure (UV15‐SnO_2_), the crystalline phase was retained without notable changes in the grain size (1.5–3 nm) or the electron diffraction ring patterns (Figure 2D). In addition to cross‐sectional TEM analysis, the topography of SnO_2_ was investigated after different UV exposure times. Figure 2E presents the atomic force microscopy (AFM) image of UV0‐SnO_2_, indicating an extremely flat surface with no discernible crystalline morphology. However, SnO_2_ nanocrystals began to appear after 5 min of UV exposure, and the interconnected small and dense SnO_2_ grains became more pronounced after 10 and 15 min (Figure 2F–H). The average surface roughness increased slightly from 0.9 to 3.4 nm after 15 min. In the X‐ray diffraction spectra, the broad humps were observed in the 2θ range of 20–40° for both UV0‐ and UV15‐SnO_2_ (Figure S1, Supporting Information). While the fully amorphous UV0‐SnO_2_ showed no distinct peaks, the (211) diffraction peak was clearly observed in UV15‐SnO_2_. Furthermore, the overlapping (110) and (101) peaks mixed with the broad hump were likely attributable to percolated nanocrystalline SnO_2_ domains embedded within the amorphous SnO_2_ matrix.^[^ ^36^, ^37^ ^]^

**Figure 3:**
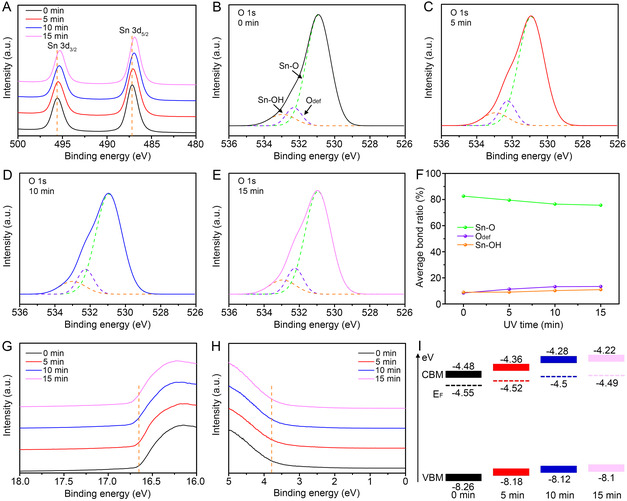
A) XPS Sn 3d spectra for UV‐SnO_2_. XPS O 1s spectra for B) UV0‐SnO_2_, C) UV5‐SnO_2_, D) UV10‐SnO_2_, and E) UV15‐SnO_2_. F) Average bond ratio plots obtained for five independent UV‐SnO_2_ thin films. UPS spectra of UV‐SnO_2_ in the G) high‐binding and H) low‐binding energy regions. I) Energy band diagrams for UV‐SnO_2_.

During the reaction between amorphous SnO_2_ and the radicals generated under UV exposure, the atomic composition and defect density changed rapidly. As shown in the X‐ray photoelectron spectroscopy (XPS) results, the Sn 3d binding energy decreased with increasing UV exposure time (**Figure** **3**A). The reduced density of undercoordinated Sn^4+^ caused by the formation of new chemical bonds with the generated radicals led to binding energy reductions from 495.59 to 495.36 eV and from 487.17 to 486.94 eV after 15 min of UV exposure.^[^ ^38^ ^]^ Furthermore, the absence of spectral shoulders indicates the presence of pure Sn^4+^. The deconvoluted O 1s spectra corresponding to the Sn–OH, Sn–O, and O_def_ species of UV0‐, UV5‐, UV10‐, and UV15‐SnO_2_ are shown in Figure 3B–E, respectively. In each case, the peaks corresponding to the Sn–OH, Sn–O, and O_def_ species were observed at 532.38, 532.25, and 530.91 eV, respectively. From integration of the spectral areas, the proportion of Sn–O decreased from 82.61% for UV0‐SnO_2_ to 75.65% for UV15‐SnO_2_ (Figure 3F). Additionally, the proportions of the O_def_ and Sn–OH species increased from 8.42 to 13.4% and from 8.97 to 10.95%, respectively. This can be explained by the fact that under UV exposure, the density of oxygen defects increased as the Sn–O bonds broke, leading to the reconstruction of the SnO_2_ matrix, accompanied by the adsorption of –OH groups on the SnO_2_ surface.^[^ ^39^ ^]^ The adsorbed O_3_/OH radicals on the outermost surface bind strongly with the dangling bonds of Sn atoms, resulting in a higher oxygen content compared to the inner regions. After etching the UV0‐SnO_2_ surface to a depth of 2 nm by Ar sputtering, the O to Sn ratio was found to be 2.05:1. In contrast, for UV15‐SnO_2_, the O to Sn ratio significantly decreased to 1.72:1 after etching to the same depth. The reduced oxygen content in the inner regions following UV exposure suggests that additional oxygen vacancies were formed during crystallization. DFT calculations revealed that the oxygen vacancy formation energies in the amorphous structure are significantly lower (1.3–3.6 eV) than in crystalline rutile SnO_2_ (4.0 eV) (Figures S2 and S3, and computational details in Supporting Information). This lower energy requirement promotes the formation of oxygen vacancies in the amorphous regions during the UV‐induced crystallization process, consistent with experimental observations of reduced oxygen content in the inner regions. Considering that diffused radicals within the SnO_2_ matrix may also remain in interstitial sites without binding, such dramatic changes in *O*_def_ can significantly affect the electronic structure and electrical properties. Figure 3G,H shows the ultraviolet photoelectron spectroscopy (UPS) results in the high‐ and low‐binding energy regimes, respectively. The increased binding energy cutoffs ($E_{\mathrm{cut}}$) and decreased Fermi edges ($E_{F,\mathrm{edge}}$) observed with longer UV exposure times suggest a significant upward shift in the energy levels owing to *O*_def_ enrichment; these results correlate with the typical *n*‐doping effects observed in semiconductors.^[^ ^40^, ^41^ ^]^ The Fermi levels ($E_{F}$), calculated using $E_{\mathrm{cut}}$− 21.2 (eV), were determined to be −4.55, −4.52, −4.5, and −4.49 eV for UV0‐, UV5‐, UV10‐, and UV15‐SnO_2_, respectively. The valance band maximum values, calculated using $E_{F}-E_{F,\mathrm{edge}}$, were determined to be −8.26, −8.18, −8.12, and −8.1 eV for UV0‐, UV5‐, UV10‐, and UV15‐SnO_2_, respectively. Considering the band gap ($E_{g}$) of UV‐SnO_2_, the conduction band minimum (CBM) values were calculated as −4.48, −4.36, −4.28, and −4.22 eV for UV0‐, UV5‐, UV10‐, and UV15‐SnO_2_, respectively (Figure S4, Supporting Information). A summary of the energy band diagrams for UV‐SnO_2_ is shown in Figure 3I.

**Figure 4:**
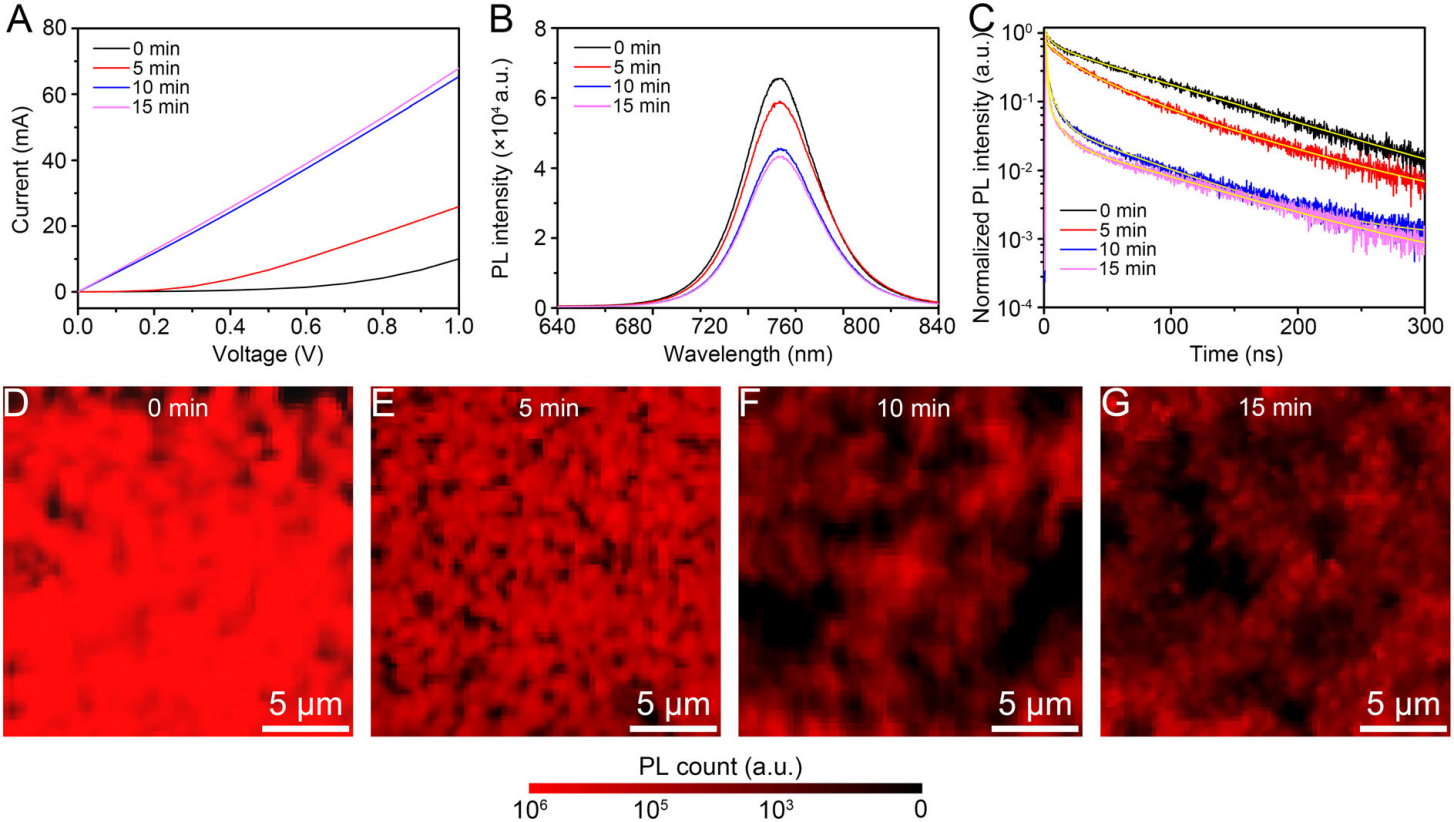
A) *I–V* curves, B) steady‐state PL spectra, and C) time‐resolved PL spectra of UV‐SnO_2_. FLIM images of D) UV0‐SnO_2_, E) UV5‐SnO_2_, F) UV10‐SnO_2_, and G) UV15‐SnO_2_.

The current–voltage (*I–V*) curves of UV‐SnO_2_ present a significant increase in electric current with increasing UV exposure time (**Figure** **4**A). The electrical conductivity (σ) was calculated using the following equation^[^ ^42^ ^]^

(1) $$\sigma =\frac{L}{\text{RA}}\;$$

where R is the resistance, and A (0.12 cm^2^) and L (10 nm) are the area and thickness of UV‐SnO_2_, respectively. Due to the low conductivity of UV0‐SnO_2_, the *I–V* profile is nonlinear in the low‐voltage region. However, since Ohmic behavior was observed beyond 0.9 V, the resistance was extracted from the region above 1 V. The conductivity values were 8.33 × 10^−5^, 2.17 × 10^−4^, 5.45 × 10^−4^, and 5.58 × 10^−4^ S cm^−1^ for UV0‐, UV5‐, UV10‐, and UV15‐SnO_2_, respectively, exhibiting excellent agreement with the average conductivities obtained from Hall effect measurements (Table S1, Supporting Information). The electron concentration increased from 9.06 × 10^11 ^cm^−3^ for UV0‐SnO_2_ to 4.37 × 10^12^ cm^−3^ for UV15‐SnO_2_, accompanied by an enhancement in the crystallinity of UV‐SnO_2_. Furthermore, this conductivity saturation observed after 10 min of UV exposure was consistent with the TEM, AFM, XPS, and UPS results. Since the main electron transport occurs through electrical percolation pathways formed by nanocrystalline SnO_2_ domains interspersed with amorphous regions, the conductivity of UV15‐SnO_2_ was lower than that of calcinated crystalline ALD‐SnO_2_ (≈1.7 S cm^−1^).^[^ ^43^ ^]^ Their enhanced conductivity and upward shift in the energy levels were directly reflected in the interfacial charge transfer behavior. To investigate this further, the carrier dynamics at the UV‐SnO_2_/perovskite interface were investigated using photoluminescence (PL) spectroscopy with a methylammonium–formamidinium mixed halide perovskite, namely (MAPbI_3_)_0.95_(FAPbIBr_2_)_0.05_. The steady‐state PL intensity of the UV10‐SnO_2_/perovskite decreased significantly to 65.9% compared to that of the UV0‐SnO_2_/perovskite at 773 nm (Figure 4B). In addition, prominent PL quenching was observed in the time‐resolved PL decay measurements after UV exposure (Figure 4C). In the early decay stage, the PL intensity decreased dramatically owing to the rapid transfer of photoexcited electrons from the CBM of the perovskite to the CBM of the SnO_2_ ETLs. This process was associated with suppressed nonradiative recombination at the interface, and it was observed that the quenching rate increased more with longer UV exposure times.^[^ ^44^, ^45^ ^]^ Furthermore, the slow rate of PL decay in the later stages was attributed to radiative recombination within the perovskite.^[^ ^44^, ^45^ ^]^ Subsequently, the decay curves were fitted using the following biexponential decay function^[^ ^44^, ^45^ ^]^

(2) $$f\left(t\right)=a_{1}e^{-t/\tau_{1}}+a_{2}e^{-t/\tau_{2}}$$

where $a_{1}$ and $a_{2}$ are the fitting parameters, and $\tau_{1}$ and $\tau_{2}$ are the times correlated with the nonradiative and radiative recombination behaviors, respectively. The average PL lifetimes were calculated as 72.57, 43.46, 14.35, and 12.38 ns for UV0‐, UV5‐, UV10‐, and UV15‐SnO_2_, respectively. This rapid decay in the PL lifetime with increasing UV exposure time can be attributed to the improved conductivity and superior band alignment between UV‐SnO_2_ and the perovskite, which facilitated electron transfer at the interface. In particular, the trap states and electron injection barrier can be reduced as the CBM alignment between the SnO_2_ ETLs and the perovskite is improved through enhanced crystallinity of UV‐SnO_2_.^[^ ^46^ ^]^ The fitting parameters and PL lifetimes are listed in Table S2, Supporting Information. The changes in the PL characteristics were further visualized using fluorescence lifetime imaging microscopy (FLIM) (Figure 4D–G). The intense red PL emission originating from the perovskite grains decreased dramatically, and broad dark regions appeared upon increasing the UV exposure time.

The surface wettability of SnO_2_ toward the perovskite solution is known to influence the kinetics of perovskite crystallization, following the heterogeneous nucleation–growth model.^[^ ^47^, ^48^, ^49^ ^]^ In the current system, perovskite deposition immediately after fabrication of the UV‐SnO_2_ ETLs led to no noticeable changes in the morphology or grain size, as the solutions efficiently wet the hydrophilic SnO_2_ surface under all UV exposure conditions (Figure S5, Supporting Information). However, 12 h after fabrication of UV0‐SnO_2_, the surface wettability decreased, giving an increased contact angle of 44.8° (Figure S6, Supporting Information). In contrast, under a prolonged storage time of 24 h, the contact angles measured for UV5‐, UV10‐, and UV15‐SnO_2_ were 9.1, 7.3, and 6.7°, respectively, suggesting that the hydrophilicity was retained (Figure S7, Supporting Information). In the XPS C 1s spectra of the same samples, the peak intensity for UV15‐SnO_2_ at 284.98 eV was 11.3% lower than that of UV0‐SnO_2_ (Figure S8, Supporting Information). This was attributed to the fact that dangling bonds within amorphous SnO_2_ formed new chemical bonds with carbon impurities in a dry atmosphere, increasing hydrophobicity, similar to the results observed for ALD‐processed metal oxides.^[^ ^22^, ^23^ ^]^ The robust nanocrystalline SnO_2_ domains passivated with a high density of hydroxyl groups resisted the adsorption of carbon impurities, thereby retaining its excellent hydrophilicity. This excellent retention of surface wettability leads to more consistent results during preparation of the perovskite layer, contributing to the reproducible fabrication of PSCs. For UV0‐SnO_2_, incomplete surface coverage was observed after deposition of the perovskite, whereas perfect surface coverage was achieved on UV15‐SnO_2_ after storage of the substrates under air for 24 h (Figure S9, Supporting Information).

**Figure 5:**
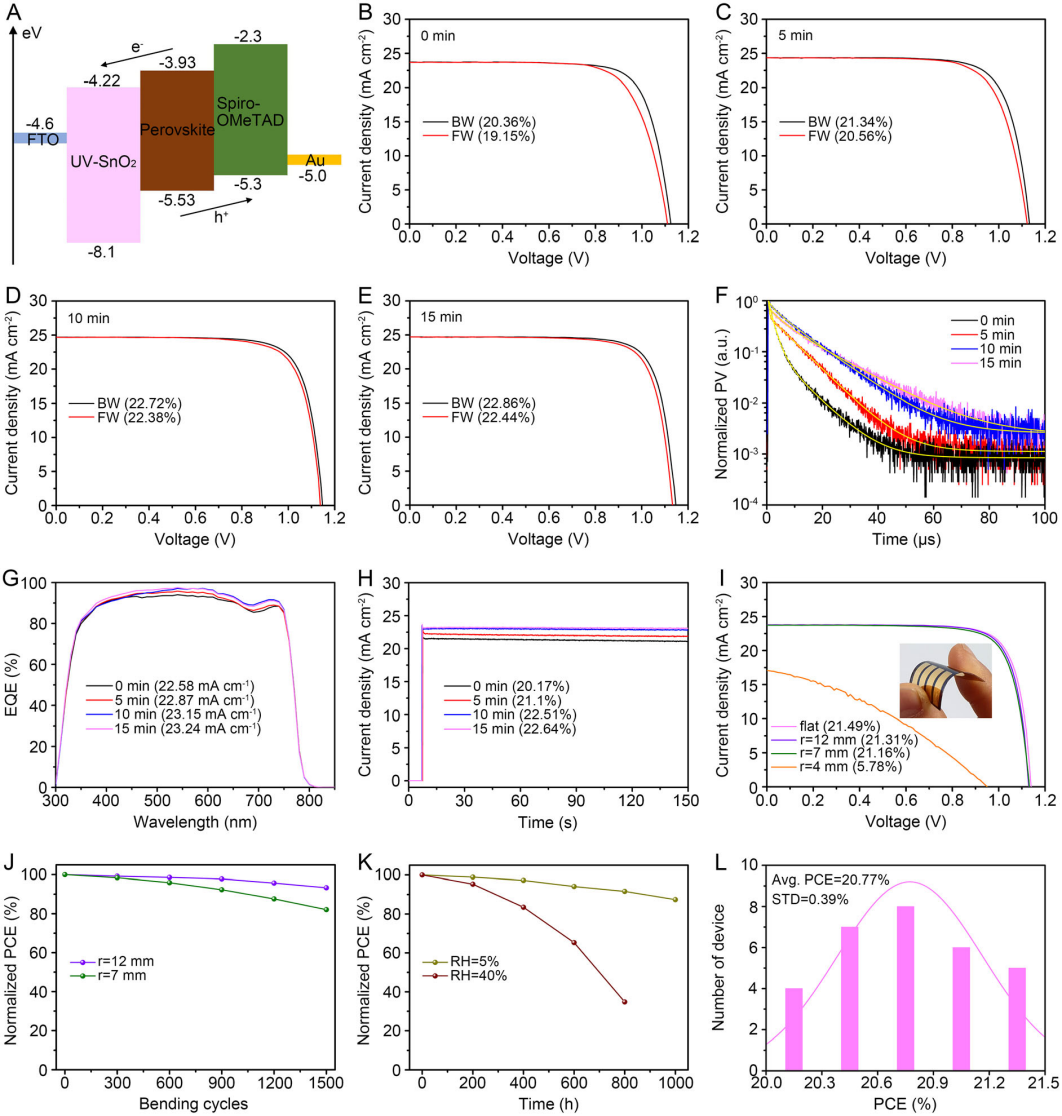
A) Energy band diagram of the PSC incorporating UV15‐SnO_2_. *J–V* curves of B) UV0 device, C) UV5 device, D) UV10 device, and E) UV15 device. F) Transient photovoltage decay curves, G) EQE spectra, and H) steady‐state photocurrent density curves of the prepared devices. I) *J–V* curves of the flexible UV15 device before bending and after bending at *r* = 12, 7, and 4 mm, with the inset showing a photograph of the bent device. J) Normalized PCE curves of the flexible UV15 devices under 1500 bending cycles at *r* = 12 mm and 7 mm. K) Normalized PCE curves of the unencapsulated flexible UV15‐devices under 1 sun illumination for 1000 h at RH = 5% and 40% (25 °C). L) PCE histogram of the flexible UV15 devices.

**Figure** **5**A illustrates the energy diagram of PSCs incorporating UV15‐SnO_2_. In this system, the photoexcited electrons and holes migrate to the fluorine‐doped SnO_2_ (FTO) and the Au electrodes through the CTLs, respectively. For the device incorporating UV0‐SnO_2_ (UV0‐device), the PCEs determined from the photocurrent density–voltage (*J–V*) curves were 20.36 and 19.15% for the backward (BW) and forward (FW) voltage sweeps, respectively (Figure 5B). After UV exposure, the resulting highly efficient ETLs led to remarkable improvements in the device performance, giving PCEs of 21.34, 22.72, and 22.86% for the BW voltage sweeps of the UV5‐, UV10‐, and UV15‐devices, respectively (Figure 5C–E). The open‐circuit voltage (*V*_oc_), short‐circuit current density (*J*_sc_), and fill factor (FF) of the UV0‐device were enhanced from 1.125 to 1.148 V, 23.72 to 24.71 mA cm^−2^, and 0.763 to 0.806, respectively, after UV exposure for 15 min. This enhancement in *V*_oc_ can be attributed to the upward shift in the CBM and Fermi level of UV‐SnO_2_, which raises the electron quasi‐Fermi level (*E*_fn_), thereby improving the band alignment and generating a larger built‐in potential.^[^ ^42^, ^50^, ^51^ ^]^ Furthermore, the conductive UV‐SnO_2_ enhanced the FF and reduced the series resistance (*R*_s_) of the devices. The *R*_s_ values were determined to be 9.7, 6.8, 3.4, and 2.9 Ω for the UV0, UV5, UV10, and UV15‐devices, respectively. Additionally, the synergetic effects resulting from the introduction of UV‐SnO_2_ were reflected in the *J–V* hysteresis behavior. Hysteresis was significantly suppressed owing to the facilitated electron injection and transfer at the UV‐SnO_2_/perovskite interfaces. With a prolonged UV exposure time of 30 min (UV30‐SnO_2_), the UV30 device retained high PCEs, exhibiting values of 22.62% and 22.32% for the BW and FW voltage sweeps, respectively (Figure S10, Supporting Information). Therefore, the observed PCE saturation after 10 min of UV exposure was consistent with completion of the crystallization process. The unchanged, pinhole‐free perovskite morphology after UV exposure of the SnO_2_ ETLs suggests that the minor increase in surface roughness of UV‐SnO_2_ is unlikely to significantly affect the defect density or charge transfer behavior within the perovskite layer. Furthermore, the inner regions remained composed of highly dense nanocrystalline domains after UV exposure, as shown in the TEM images. Compared with the PCE of ≈ 19% achieved in our previous study using spin‐coated colloidal SnO_2_ ETLs (roughness = 1.32 nm on glass/ITO), the application of UV exposure to ALD processed SnO_2_ ETLs clearly demonstrates the potential of this methodology to enhance device performance.^[^ ^52^ ^]^ The devices employing conventional SnO_2_ ETLs prepared through a sol‐gel and thermal annealing process exhibited a lower PCE of 18.36% (Figure S11, Supporting Information). Moreover, the ALD process enables conformal SnO_2_ deposition on rough FTO surfaces, whereas solution‐processed SnO_2_ ETLs often suffer from incomplete coverage, necessitating the additional deposition of passivation layers.^[^ ^15^, ^16^ ^]^

The charge recombination behavior within the devices was further investigated using transient photovoltage (TPV) analysis (Figure 5F). While the UV0 device exhibited a rapid photovoltage decay, the PV lifetimes of the other devices were extended with longer UV exposure times. The PV lifetimes were calculated as 3.98, 6.72, 10.84, and 11.43 μs for the UV0‐, UV5‐, UV10‐, and UV15‐devices, respectively (Table S2, Supporting Information). This prolonged PV lifetime indicates suppressed charge recombination at the UV‐SnO_2_/perovskite interfaces, contributing to the enhancement of all device parameters. The average PCE value obtained from 30 independent UV15‐devices was 22.27%, thereby demonstrating the high reproducibility of the SnO_2_ ALD/crystallization process (Figure S12, Supporting Information). For both thin and thick UV15‐SnO_2_ layers (i.e., 5 and 15 nm), lower PCEs of 18.7 and 20.02% were observed, respectively, along with severe *J–V* hysteresis (Figure S13, Supporting Information). Therefore, the optimal thickness of UV‐SnO_2_ was determined to be 10 nm. The effect of UV‐SnO2 thickness on device performance was considered to be governed by the trade‐off between hole blocking and the electron transfer efficiency. Thin ETLs fail to completely block diffused holes because of tunneling effects, whereas thick ETLs hinder electron transfer by reducing the mean free path of the electrons.^[^ ^53^ ^]^ Figure 5G presents the external quantum efficiency (EQE) spectra of the devices. The high EQE values of 85%–95% were retained across the 400–750 nm wavelength range. Notably, the UV10‐ and UV15‐devices exhibited enhanced EQE values in the long‐wavelength region. The absence of noticeable changes in the transmittance, absorption, and reflectance spectra of UV‐SnO_2_ on glass/FTO substrates above 300 nm suggests that variations in the parasitic optical losses do not need to be considered in the EQE spectra (Figure S14, Supporting Information). Instead, the reduced photocurrents observed in the UV0‐device can be attributed to suppressed electron injection and transfer due to charge recombination at the UV0‐SnO_2_/perovskite interfaces.^[^ ^54^, ^55^, ^56^ ^]^ The integrated J_sc_ values were determined to be 22.58, 22.87, 23.15, and 23.24 mA cm^−2^ for the UV0‐, UV5‐, UV10‐, and UV15‐devices, respectively, exhibiting excellent agreement with the *J*_sc_ values measured from the *J–V* sweeps. At the maximum power point voltages under 1 sun illumination, the PCEs were calculated as 20.17, 21.1, 22.51, and 22.64% for the UV0‐, UV5‐, UV10‐, and UV15 devices, respectively (Figure 5H). These results confirm the excellent device performance and photostability achieved under real operating conditions.

Owing to the advantages of this low‐temperature process, it was possible to fabricate highly efficient and flexible PSCs on poly(ethylene naphthalate) (PEN)/tin‐doped indium oxide (ITO) substrates using the protocol employed for the rigid devices. Despite the high‐power UV exposure, the temperature was elevated to 110–120 °C after 15 min (Figure S15, Supporting Information). Notably, this processing temperature is sufficiently mild to allow the fabrication of flexible devices on plastic substrates.^[^ ^57^ ^]^ The PEN/ITO substrates exhibited no noticeable changes in flexibility and resistance after annealing at 125 °C for 1 h in an isolated chamber (Figure S16, Supporting Information). Prior to fabricating flexible PSCs, we further investigated the effect of processing temperature on device performance using a UV illuminator consisting of an LED with the same intensity and wavelength. During UV exposure, the temperature of the PEN/ITO/UV‐SnO_2_ substrates was maintained at 34 °C, as confirmed by thermal images captured using an IR thermal imaging camera (Figure S17, Supporting Information). The UV15‐device exhibited excellent PCEs of 22.38% and 22.03% for the BW and FW voltage sweeps, respectively, comparable to those of the device employing UV15‐SnO_2_ prepared using an Hg lamp‐based UV illuminator (Figure S18, Supporting Information). Therefore, only the effect of UV exposure was considered in the fabrication of flexible devices. Figure 5I presents the *J–V* curves of the flexible UV15‐device, measured before and after bending at radii (r) of 12, 7, and 4 mm. Prior to bending, the PCE was 21.49%, closely approaching that of the corresponding rigid device. After bending to *r* = 12 and 7 mm, the PCE decreased slightly to 21.31 and 21.16%, respectively. Although the Young's modulus of the SnO_2_ ETLs may slightly increase during crystallization, the percolation networks formed by nanocrystalline SnO_2_ domains help relieve bending stress at their junctions, thereby retaining mechanical stability until the ITO layers fracture.^[^ ^58^ ^]^ Upon bending to *r* = 4 mm, the device ruptured owing to high bending stress, which fractured the ITO layers and led to electrical failure (Figure S19, Supporting Information).^[^ ^59^, ^60^, ^61^ ^]^ The released energy propagated directly to the UV15‐SnO_2_ and perovskite layers, leading to their fracture and device failure (PCE = 5.78%) (Figure S20, Supporting Information). However, at *r* = 12 and 7 mm, the devices demonstrated excellent PCE retention, maintaining 93.3 and 82.1% of their initial values after 1500 bending cycles, respectively (Figure 5J). The greater reduction in the normalized PCE at *r* = 7 mm can be attributed to plastic deformation of the PEN substrate caused by mechanical yielding. Additionally, the unencapsulated UV‐15 device exhibited a remarkable photostability, retaining 87.4% of its initial PCE after 1000 h under 1 sun illumination at 25 °C and RH of 5% (Figure 5K). At a higher RH of 40%, degradation of the perovskite was accelerated, leading to device failure after 800 h. This result followed a trend similar to that reported for unencapsulated PSCs under these conditions.^[^ ^62^, ^63^ ^]^ The reproducibility of the fabrication process was confirmed in flexible devices. In the PCE histogram recorded for 30 independent devices, all devices exceeded a PCE of 20%, with an impressive average PCE of 20.77% and a standard deviation (STD) of 0.39% (Figure 5L).

## 3 Conclusions

In summary, highly flexible and efficient PSCs were designed by applying high‐power UV exposure to ALD‐processed amorphous SnO_2_. The dense O_3_/OH radicals generated upon UV exposure effectively ruptured the atomic bonds within the SnO_2_ matrix, facilitating lattice rearrangement and crystallization. This photocrystallization process enhanced the electrical conductivity and induced an upward shift in the energy levels of UV‐SnO_2_. As a result, the PCE was significantly enhanced from 20.36 to 22.86% after 15 min of UV exposure, driven by promoted electron injection and transfer at the UV‐SnO_2_/perovskite interface. Furthermore, the low‐temperature deposition and crystallization of SnO_2_ enabled the reproducible fabrication of flexible devices. Notably, a flexible UV15‐device demonstrated an outstanding PCE of 21.49% before bending and retained 93.3% of its initial value after 1500 bending cycles at *r* = 12 mm. In addition to solar cells, the low‐temperature photocrystallization process can potentially be extended to the fabrication of metal oxide‐based flexible ferroelectric memories, field‐effect transistors, and chemical sensors. By taking advantage of the ability to expose UV light uniformly over a large area, we believe it is possible to fabricate reproducible, large‐scale devices.

## 4 Experimental Section

4.1

4.1.1

##### Materials

Lead iodide (PbI_2_, 99.999%), lead bromide (PbBr_2_, 99.999%), Li bis (trifluoromethylsulfonyl)imide (Li–TFSI) (>98%), anhydrous chlorobenzene (CB, 99.8%) anhydrous dimethylformamide (DMF, 99.8%), anhydrous dimethylsulfoxide (DMSO, 99.9%), 4‐*tert*‐butylpyridine (*t*BP, 98%), and anhydrous butyl acetate (BA, >99%) were purchased from Sigma Aldrich. Methylammonium iodide (MAI, 99.8%), methylammonium bromide (99.8%), and formamidinium iodide (FAI, 99.8%) were purchased from GreatCell Solar. Tetrakis(dimethylamino)tin(IV) (TDMASn) (99%) was purchased from Strem Chemicals. Tin (II) chloride hydrate (99.995%) was purchased from Thermo Fisher Scientific. Spiro‐OMeTAD (99.5%) was purchased from LumTec. Spiro‐OMeTAD solutions were prepared by dissolving 56 mg of spiro–OMeTAD, 5.6 mg of Li‐TFSI, and 28 μl of *t*BP in 1 mL of CB. The MAPbI_3_ precursor solution was prepared by dissolving 461 mg of PbI_2_, 159 mg of MAI, and 78 mg of DMSO in 600 mg of DMF. The FAPbIBr_2_ precursor solution was prepared by dissolving 367 mg of PbBr_2_, 172 mg of FAI, and 78 mg of DMSO in 600 mg of DMF. Subsequently, 200 μL of MAPbI_3_ and 10 μL of FAPbIBr_2_ solutions were mixed and used for the deposition of the (MAPbI_3_)_0.95_(FAPbIBr_2_)_0.05_ films.

##### Fabrication of SnO2 Thin Films

SnO_2_ thin films were deposited on glass/FTO and PEN/ITO substrates by ALD using a commercial thermal ALD reactor (Savannah S200, Veeco Instruments Inc.). The chamber was kept at 125 °C under a flow of N_2_ (90 sccm) to maintain a pressure of 0.45 Torr throughout the deposition. TDMASn kept at 65 °C was used as the Sn precursor, while deionized water was used as the oxygen precursor. The full ALD cycle consisted of the following sequence: TDMASn pulse (0.8 s) ‐ N_2_ purge (10 s) ‐ H_2_O pulse (0.015 s) ‐ N_2_ purge (10 s). A growth rate of 0.63 Å/cycle was obtained based on this process, which was repeated as appropriate to obtain the desired film thickness. The SnO_2_ ETLs processed via the sol–gel method were prepared using a Sn precursor solution. Tin chloride hydrate (0.1 M) was dissolved in anhydrous ethanol, and the resulting solution was spin coated onto glass/FTO substrates at 3000 rpm for 30 s. The substrates were then annealed at 185 °C for 3 h in air.^[^ ^64^ ^]^

##### Fabrication of Solar Cells

Patterned glass/FTO (Pilkington) or PEN/ITO (Fine Chemicals Industry) substrates were ultrasonicated in a mixed solution of 2‐propanol and acetone (v/v = 1:1) for 10 min. The substrates were then rinsed thoroughly with fresh deionized water and dried under a N_2_ atmosphere. After deposition of SnO_2_ thin films using ALD, the substrates were exposed to UV light (400 mW cm^−2^/320–400 nm, JECO‐UV Machine) 5–15 min. The irradiation distance was fixed at 30 mm from the light source. The perovskite solution was then spin coated onto the UV‐SnO_2_ ETLs in two steps: 1000 rpm for 5 s and 4000 rpm for 15 s. At the second step, 69 μL of BA was dripped on the rotating substrate during the remaining 7 s. The transparent samples were immediately annealed at 65 °C for 1 min and then at 135 °C for 3 min to complete the perovskite crystallization. After cooling the substrates to room temperature, spiro‐OMeTAD solutions were spin coated onto the perovskite at 2500 rpm for 20 s. Au contacts (80 nm) were deposited using a thermal evaporator at a constant rate of 0.5 Å s^−1^.

##### Characterization

Cross‐sectional TEM images were obtained using an FEI TitanTM 80‐300 instrument. To prepare the TEM samples, UV‐SnO_2_ was deposited on a Si wafer, followed by the deposition of Au thin films to protect UV‐SnO_2_ from damage caused by the focused ion beam (Helios G4 HX). Field‐emission scanning electron microscopy images were acquired using a JSM‐7600 F (JEOL). The transmittance, absorption, and reflectance spectra of UV‐SnO_2_ were measured using a UV–VIS–NIR spectrophotometer (SHIMADZU, UV‐3600) equipped with an integrating sphere (SHIMADZU, MPC‐3100). Glass/FTO substrates were used as the background reference during the UV–VIS spectroscopy analysis. XPS and UPS were performed using an X‐ray photoelectron spectrometer (Nexsa; Thermo Fisher Scientific). The surface morphologies of the samples were analyzed by AFM (XE‐7, Park Systems). The steady‐state PL spectra and FLIM images were obtained using a spectrometer (XperRAM, Nanobase) equipped with an optical lens. The time‐resolved PL decay curves were obtained using a picosecond‐pulsed diode laser (405 nm) and a time‐correlated single‐photon counting system (XperRAM, Nanobase). Current–voltage (*I–V*), photocurrent density–voltage (*J–V*), and steady‐state photocurrent curves were obtained using a Keithley 2400 source meter. A 150 W arc xenon lamp in a solar simulator was used as the light source (Peccell). The light intensity was adjusted to AM 1.5 G using an NREL‐calibrated Si solar cell equipped with a KG‐1 filter. The active area (0.12 cm^2^) was defined using metal masks. EQE spectra were obtained at wavelengths of 300–850 nm using a QuantX 300 spectrophotometer (Oriel). TPV measurements were performed using a nanosecond laser (10 Hz, NT342A, EKSPLA) as the small‐perturbation light source and a Xe lamp (300 W, Newport) as the bias light source. The device was directly connected to a digital oscilloscope (350 MHz, MDO4034C, Tektronix) with the input impedance set to 1 MΩ. A strong attenuated laser pulse (550 nm) was used to generate a transient voltage (Δ*V*) of <20 mV.

## Conflict of Interest

The authors declare no conflicts of interest.

## Supporting information

Supplementary Material

## Data Availability

The data that support the findings of this study are available from the corresponding author upon reasonable request.
