# Supplementary material for: Low‐Temperature Photocrystallization of Atomic Layer Deposition‐Processed Tin Oxide for Highly Efficient and Flexible Perovskite Solar Cells
Source: Small Sci. 2025 Jul 7;5(9):2500196. doi: 10.1002/smsc.202500196 (PMC12412567; doi:10.1002/smsc.202500196)
Supplement: Supplementary file 1 — Supplementary Material [file SMSC-5-2500196-s001.pdf]

Supporting Information

Low-Temperature Photocrystallization of ALD-Processed Tin Oxide for Highly Efficient and Flexible Perovskite Solar Cells

Dayeon Ko[1], Se Hun Joo[1], Sol Kim[1,2], In Soo Kim[2*], Minwoo Park[1*]

[1]Department of Chemical and Biological Engineering, Sookmyung Women's University, Seoul 04310, Korea

[2]Nanophotonics Research Center, Korea Institute of Science and Technology (KIST), Seoul, 02792, Korea

*Corresponding author e-mail: isk@kist.re.kr, mwpark@sm.ac.kr

Calculation Details of OH Radical and O_3 Concentrations

Experimental Conditions

1. Wavelength: UVA (320 nm – 400 nm)
2. Light intensity: 400 mW cm^{-2}
3. Air Composition: 21% O_2, trace NO_x, H_2O vapor (RH=30% & 50%)

In typical ambient air with no added precursors, the formation of OH and O_3 from UVA exposure is generally low and occurs primarily through by secondary mechanisms, which mainly involve:

- NO_2 photolysis (but needs <400 nm, effective ~300-420 nm)
- H_2O + excited organics
- Photocatalytic-driven reactions via aromatic volatile organic compounds (VOCs) or surface catalysis

We adopted empirically determined values for radical formation rates from published literature, selected to match our experimental conditions.

OH radical: ~10^5 – 10^7 molecules $cm^{-3}s^{-1}$[S1, S2]

O3: ~1 – 10 ppb min^{-1} depending on VOCs[S3, S4]

UVA Irradiation Time (t)

- 0, 5, 10, 15, 20 min

Assumptions for Calculations

- OH generation rate:

RH (%)	Estimated OH Production Rate
30	0.7×10^6 molecules $cm^{-3}s^{-1}$
50	1.0×10^6 molecules $cm^{-3}s^{-1}$

- O_3 generation rate: 5 ppb min^{-1}*

  *assumed for both RH 30 and 50% owing to negligible dependence on RH

Concentration of OH radicals:

(10^6 molecules $cm^{-3}s^{-1}$) × (60 s min^{-1}) = 6 × 10^7 molecules $cm^{-3}min^{-1}$

[OH radical] × (t) = 6 × 10^7 molecules cm^{-3} × t

t (min)	Conc. of OH Radicals (molecules cm^{-3})	
	RH=30%	RH=50%
0	0	0
5	2.1 × 10^8	3 × 10^8
10	4.2 × 10^8	6 × 10^8
15	6.3 × 10^8	9 × 10^8
20	8.4 × 10^9	1.2 × 10^9

Concentration of O_3:

[O_3] × (t) = 5 ppb min^{-1} × t

t (min)	Conc. of O_3 (ppb)	
	RH=30%	RH=50%
0	0	0
5	25	25
10	50	50
15	75	75
20	100	100

[S1] C. George, M. Ammann, B. D'Anna, D. J. Donaldson, S. A. Nizkorodov, *Chem. Rev.* **2015**, *115*, 3218-4258.

[S2] R. R. Dickerson, D. H. Stedman, A. C. Delany, *J. Geophys. Res.* **1982,** *87*, 4933-4946.

[S3] H. Witkowski, W. Jackiewicz-Rek, J. Jaroslawski, K. Chilmon, A. Szkop, *Materials* **2022**, *15*, 5905.

[S4] R. Atkinson, J. Arey, *Chem. Rev.* **2003**, *103*, 4605-4638.

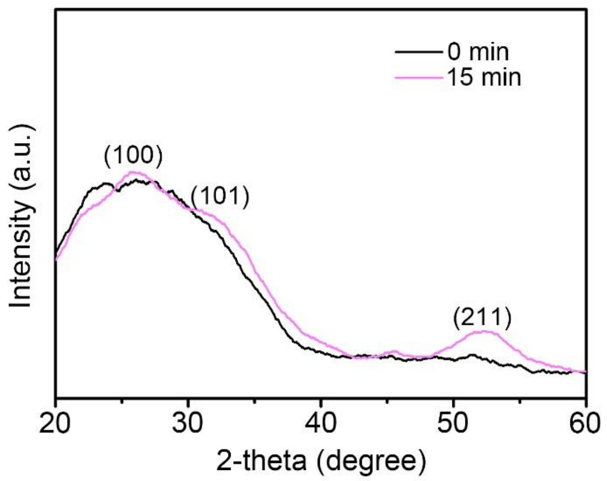

Figure S1. XRD patterns of UV0- and UV15-SnO_2.

Computational Details

To model amorphous tin oxide grown by atomic layer deposition, we generated random structures with a chemical composition of $SnO_{1.72}$ using ab initio random structure search (AIRSS).[S5] To generate random sensible structures, minimum atomic separations were set to 2.61 Å, 2.06 Å, and 3.21 Å for O-O, O-Sn, and Sn-Sn pairs, respectively, and the number of symmetry operations was randomly chosen as either 1 or 2. We then constructed a unit cell with a volume of 146 $Å^3$, containing four Sn atoms and seven O atoms randomly distributed within the cell. Initially, 30 random structures were generated and optimized using spin-polarized density functional theory (DFT) calculations implemented in CASTEP with the PBESOL exchange-correlation functional, QC5 on-the-fly pseudopotentials, a 340 eV plane wave cutoff, and a Monkhorst-Pack k-point grid with a spacing of $0.07 \times 2\pi$ $Å^{-1}$.[S6,S7] The resulting local minima structures were further refined using the more accurate RSCAN functional and C19 on-the-fly pseudopotentials with an increased plane wave cutoff of 630 eV.[S8] Brillouin zone integration was performed using a Monkhorst-Pack k-point grid with a spacing of $0.05 \times 2\pi$ $Å^{-1}$. The convergence criteria were set to 5.0×10^{-7} eV for electronic minimization, 5.0×10^{-6} eV for energy change, 1.0×10^{-2} eV/Å for maximum force, and 5.0×10^{-4} Å for atomic displacement during geometry optimization. From these refined structures, five low-energy unique structures were selected to investigate oxygen vacancy formation (**Figure S2**). For each model, three distinct defect configurations were created by removing one oxygen atom at random, followed by full structural optimization. The oxygen vacancy formation energies (E_{O_v}) were calculated according to the equation below (**Figure S3**).

$$E_{O_v} = E_{defective} + \frac{1}{2}E_{O_2} - E_{pristine}$$

where $E_{pristine}$ represents the total energy of the pristine unit cell, $E_{defective}$ denotes the total energy of the unit cell containing a single oxygen vacancy, and E_{O_2} corresponds to the total energy of an O_2 gas molecule in its ground state.

[S5] C. J. Pickard, R. J. Needs, *J. Phys.: Condens. Matter* **2011**, *23,* 053201.

[S6] S. J. Clark, M. D. Segall, C. J. Pickard, P. J. Hasnip, M. I. J. Probert, K. Refson, M. C. Payne, *Z. Kristallog.* **2005**, *220,* 567-570

[S7] J. P. Perdew, A. Ruzsinszky, G. I. Csonka, O. A. Vydrov, G. E. Scuseria, L. A. Constantin, X. Zhou, K. Burke, *Phys. Rev. Lett.* **2008**, *100,* 136406.

[S8] A. P. Bartók, J. R. Yates, *J. Chem. Phys.* **2019**, *150,* 161101.

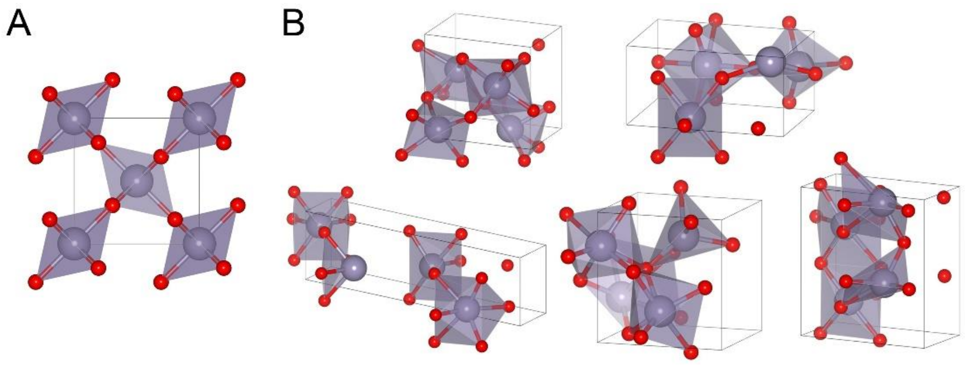

Figure S2. Optimized atomic structures of (A) crystalline and (B) amorphous tin oxide. The amorphous configurations were generated through ab initio random structure search. Violet and red spheres represent Sn and O atoms, respectively. The crystalline structure exhibits perfect octahedral coordination, while amorphous structures show diverse local environments.

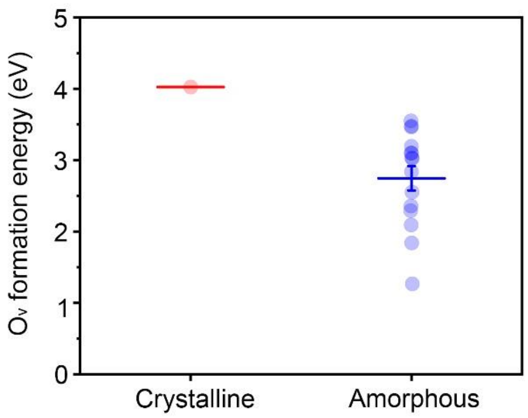

Figure S3. Comparison of calculated oxygen vacancy formation energies in crystalline and amorphous tin oxide. Horizontal lines indicate the average values (4.0 eV for crystalline and 2.7 eV for amorphous), while individual data points represent different vacancy configurations.

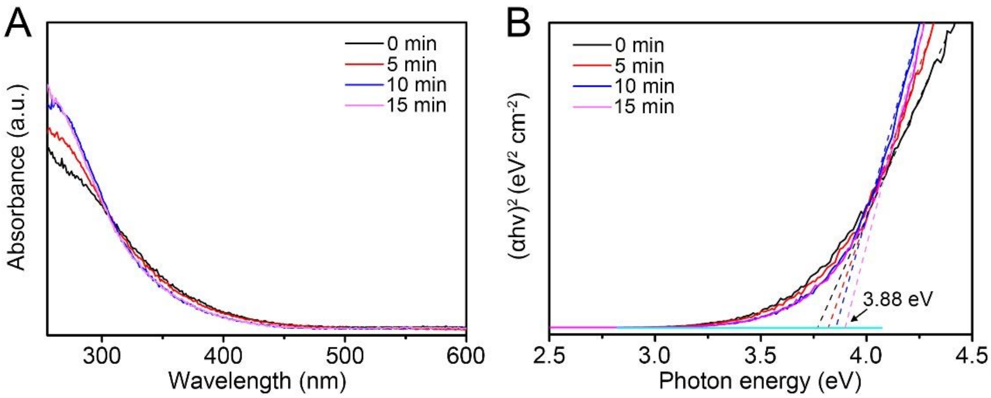

Figure S4. (A) UV-visible spectra and (B) Tauc plots of UV-SnO_2. The bandgap values were calculated as 3.78, 3.82, 3.84, and 3.88 eV for UV0, UV5, UV10, and UV15-SnO_2, respectively.

Table S1. Electrical properties of UV-SnO_2.

Samples	Conductivity [S cm^{-1}][a)]	Conductivity [S cm^{-1}][b)]	Electron concentration [cm^{-3}][b)]
UV0-SnO_2	8.33×10^{-5}	8.57×10^{-5}	9.06×10^{11}
UV5-SnO_2	2.17×10^{-4}	2.28×10^{-4}	2.01×10^{12}
UV10-SnO_2	5.45×10^{-4}	5.22×10^{-4}	4.28×10^{12}
UV15-SnO_2	5.58×10^{-4}	5.39×10^{-4}	4.37×10^{12}

[a)]The values were calculated from I–V curves.

[b)]The average values from five independent samples were obtained using a Hall effect measurement system.

Table S2. Time-resolved photoluminescence (TRPL) parameters of the UV-SnO_2/perovskite fitted using a bi-exponential decay function.[a)]

Sample	a_1	τ_1 [ns]	a_2	τ_2 [ns]	τ_{avg} [ns] [b)]
UV0-SnO_2	0.516	47.36	0.484	99.46	72.57
UV5-SnO_2	0.754	25.48	0.246	98.55	43.46
UV10-SnO_2	0.902	6.73	0.098	84.47	14.35
UV15-SnO_2	0.928	5.36	0.072	102.8	12.38

[a)]Fit function = $a_1 e^{-t/\tau_1} + a_2 e^{-t/\tau_2}$

[b)]$\tau_{avg} = (\sum_i a_i \tau_i)/(\sum_i a_i)$, where $\sum_i a_i = 1$

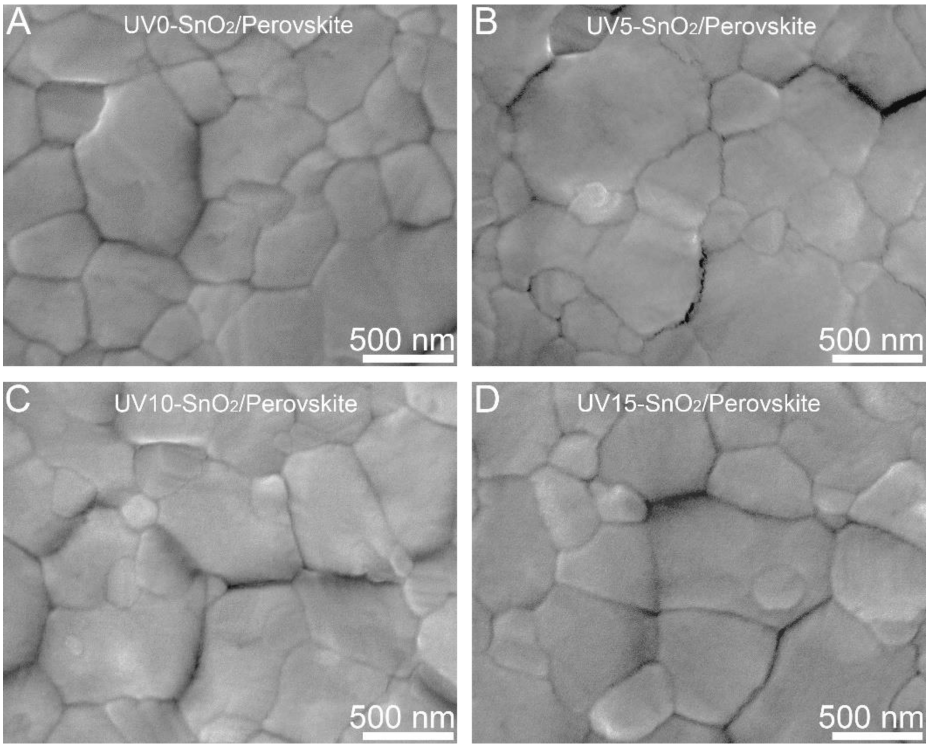

Figure S5. SEM images of the (A) FTO/UV0-SnO_2/perovskite, (B) FTO/UV5-SnO_2/perovskite, (C) FTO/UV10-SnO_2/perovskite, and (D) FTO/UV15-SnO_2/perovskite. The perovskites were deposited immediately after the fabrication of the UV-SnO_2 subsrates.

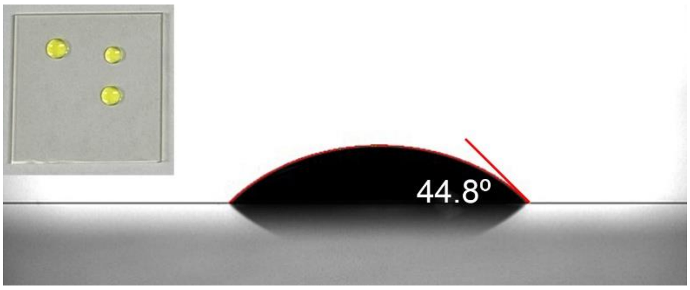

Figure S6. Photographic image of the contact angle measurement upon dropping the perovskite precursor on the glass/UV0-SnO_2 substrate. The inset photograph highlights the poor wettability of the droplets. The precursor solution was dropped onto the surface 12 h after UV0-SnO_2 fabrication, during which time the substrate was stored under air at 25 °C and 5% RH.

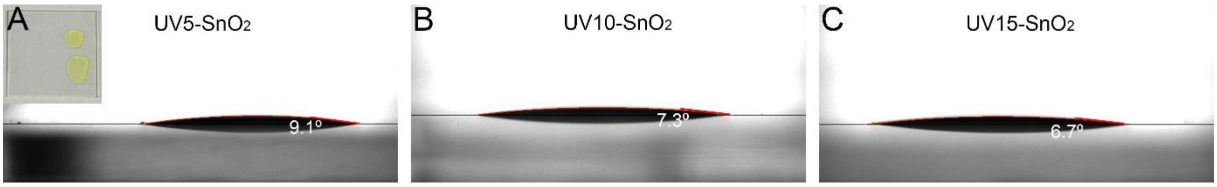

Figure S7. Photographic images of the contact angle measurement upon dropping the perovskite precursor on the (A) glass/UV5-SnO_2. (B) glass/UV10-SnO_2, and (C) glass/UV15-SnO_2 substrates. The inset image in (A) highlights the high wettability of the surface. The precursor solutions were dropped onto the surface 24 h after UV5-SnO_2, UV10-SnO_2, and UV15-SnO_2 fabrication, during which time the substrate was stored under air at 25 ºC and 5% RH.

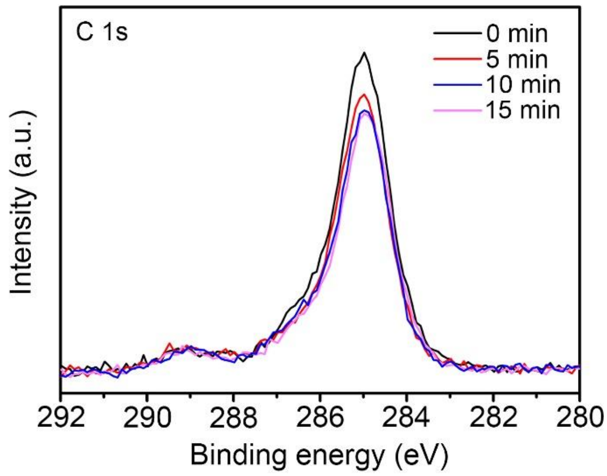

Figure S8. XPS C 1s spectra for UV-SnO_2. Prior to XPS analysis, UV0-SnO_2 was stored under air at 25 °C and 5% RH for 12 h, while the UV5-SnO_2, UV10-SnO_2, and UV15-SnO_2 substrates were stored under the same conditions for 24 h.

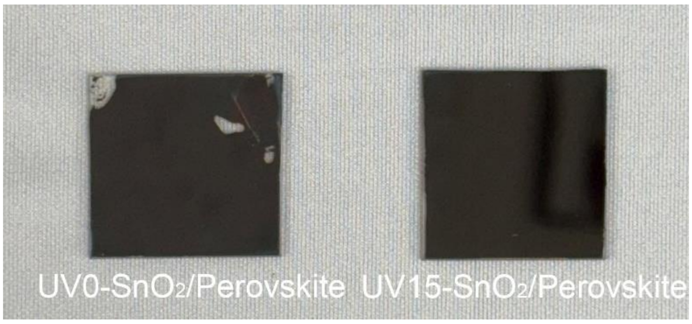

Figure S9. Photographic image of the glass/UV0-SnO_2/perovskite and glass/UV15-SnO_2/perovskite. Prior to perovskite deposition, the glass/UV0-SnO_2 and glass/UV15-SnO_2 substrates were stored under air at 25 °C and 5% RH for 24 h.

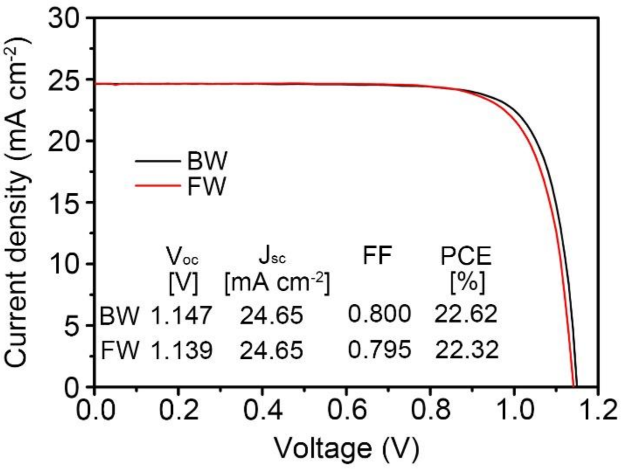

Figure S10. J–V curves of the UV-30 device.

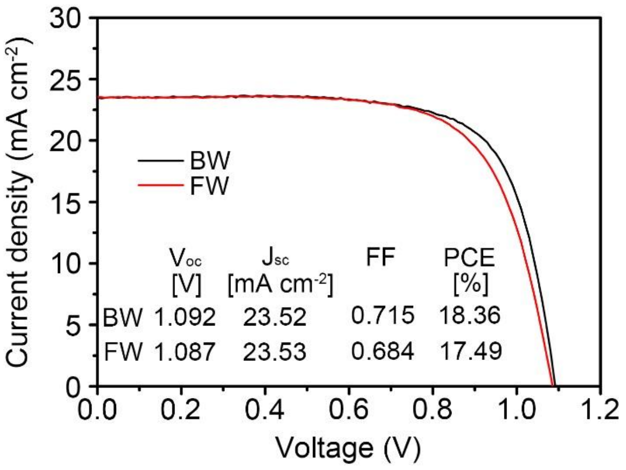

Figure S11. J–V curves of the PSC employing sol-gel processed SnO_2 ETLs.

Table S2. TPV parameters of the complete devices incorporating UV-SnO_2 fitted using a bi-exponential decay function.[a)]

Device	a_1	τ_1 [μs]	a_2	τ_2 [μs]	τ_{avg} [μs] [b)]
UV0-device	0.495	1.34	0.505	6.56	3.98
UV5-device	0.387	1.78	0.613	9.84	6.72
UV10-device	0.291	2.85	0.709	14.12	10.84
UV15-device	0.308	2.62	0.692	15.35	11.43

[a)]Fit function = $a_1 e^{-t/\tau_1} + a_2 e^{-t/\tau_2}$

[b)]$\tau_{avg} = (\sum_i a_i \tau_i)/(\sum_i a_i)$, where $\sum_i a_i = 1$

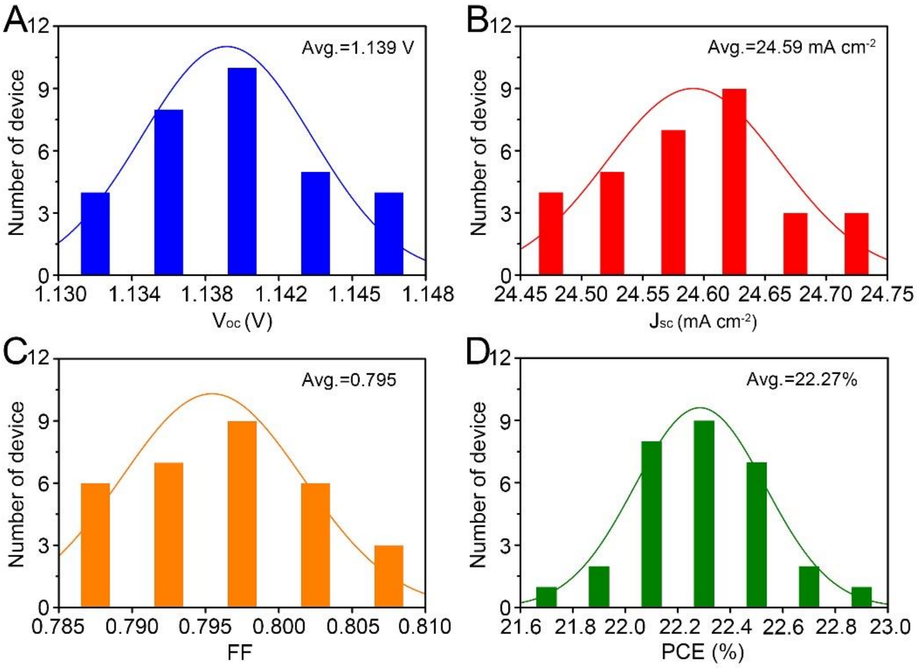

Figure S12. Histograms of (A) V_{oc}, (B) J_{sc}, (C) FF, and (D) PCE for 30 independent rigid UV15-devices.

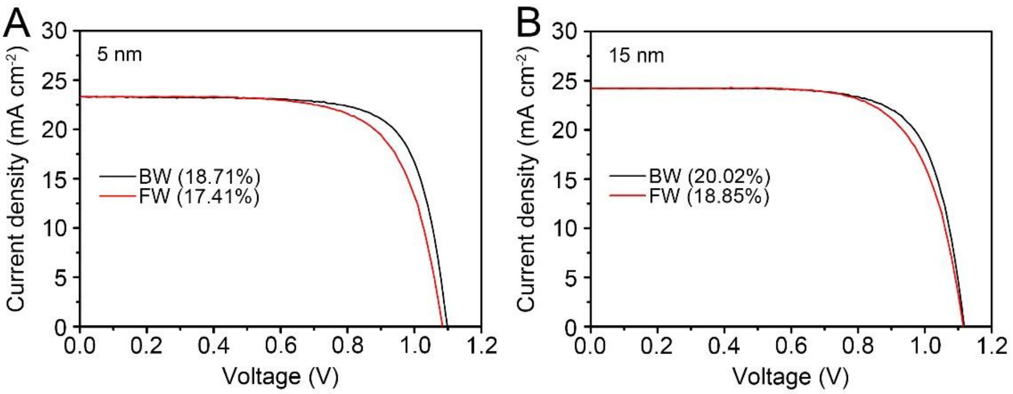

Figure S13. J-V curves of the rigid devices incorporating UV15-SnO_2 with thicknesses of (A) 5 nm and (B) 15 nm.

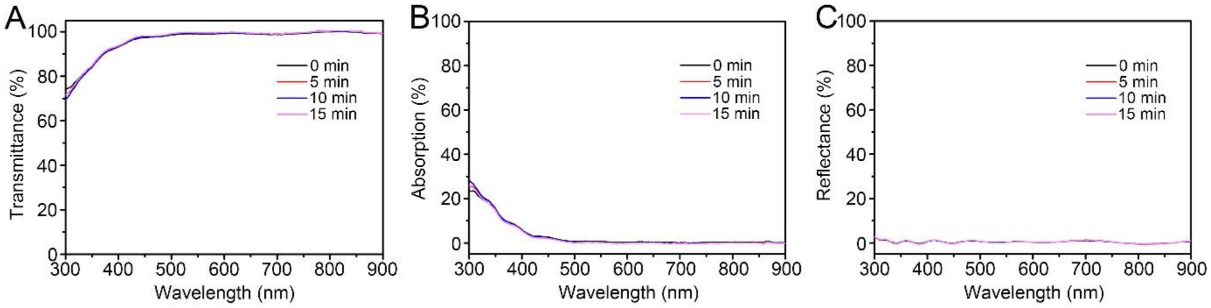

Figure S14. (A) Transmittance, (B) absorption, and (C) reflectance spectra of UV-SnO_2 on glass/FTO substrates.

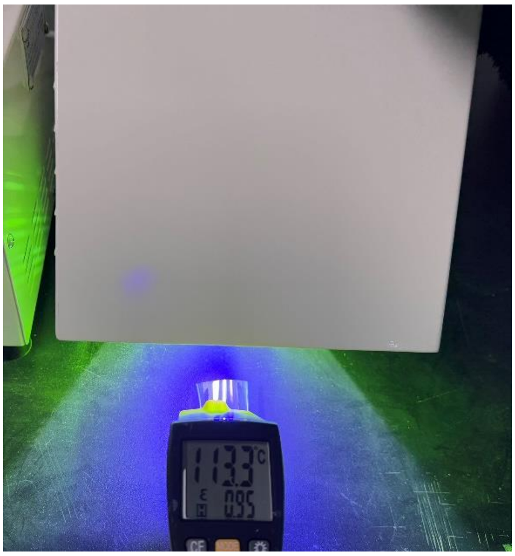

Figure S15. Photographic image showing the temperature of the PEN/ITO substrate after 15 min of UV light illumination using an Hg lamp.

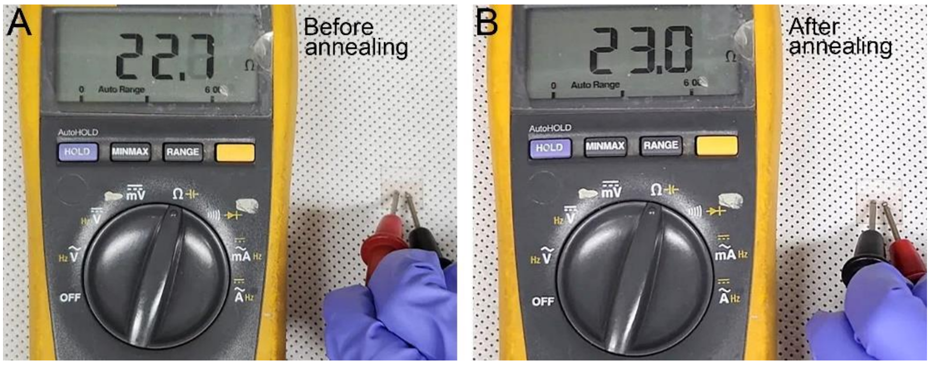

Figure S16. Photographic images of the PEN/ITO substrate: (A) before annealing and (B) after annealing at 125 °C for 1 h in an isolated chamber.

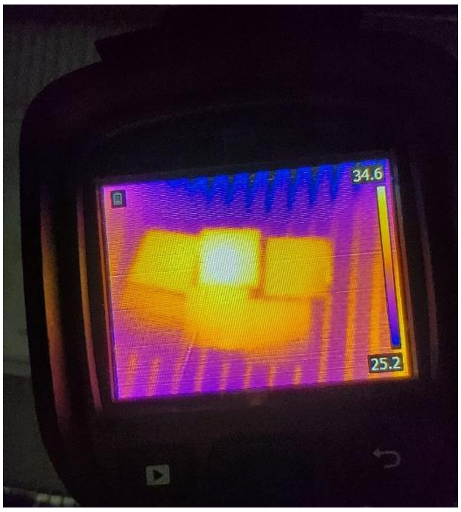

Figure S17. Photographic image showing the temperature of the PEN/ITO substrates after UV exposure for 15 min using an LED.

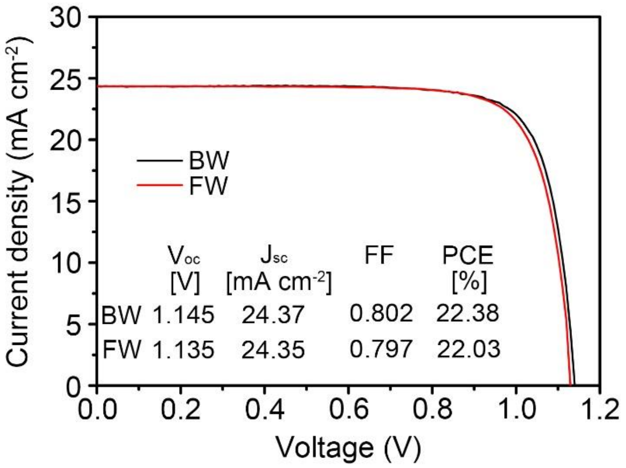

Figure S18. J–V curves of the UV15-device. UV15-SnO_2 was prepared using an LED-based UV illuminator with the same intensity and wavelength.

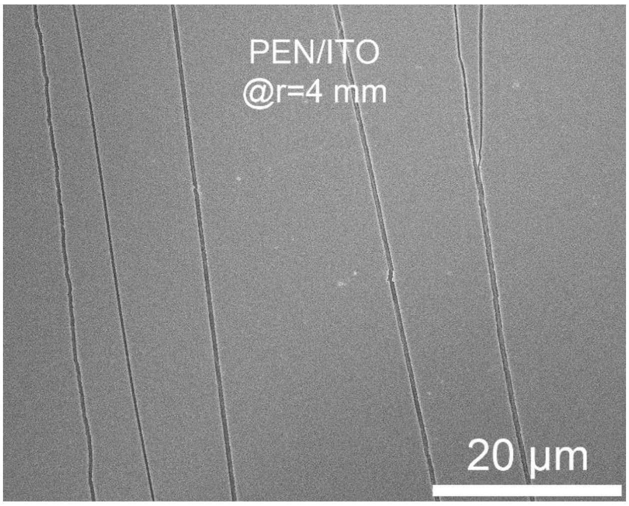

Figure S19. SEM image of a PEN/ITO substrate at r = 4 mm.

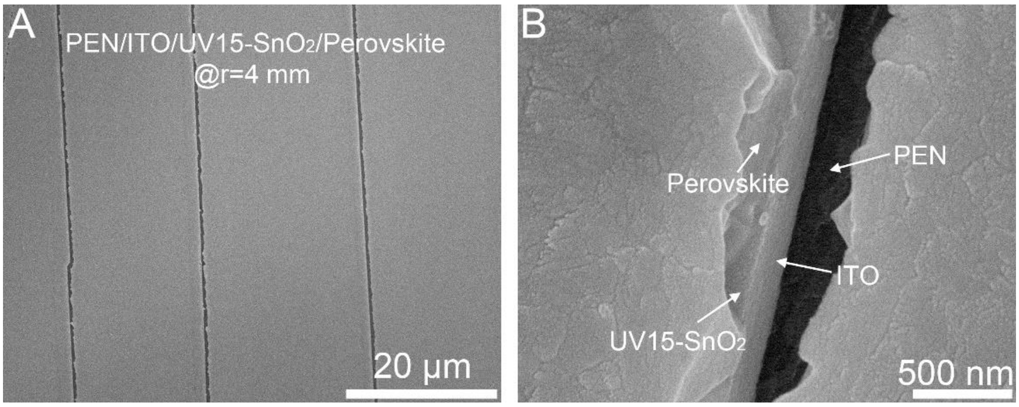

Figure S20. (A) SEM image of the PEN/ITO/UV15-SnO_2/perovskite at r = 4 mm. (B) Magnified and tilted SEM image corresponding to that presented in (A).
